# Effects of early recombinant human growth hormone treatment in young Chinese children with Prader–Willi syndrome

**DOI:** 10.1186/s13023-023-02615-7

**Published:** 2023-02-07

**Authors:** Ying Gao, Li-Li Yang, Yang-Li Dai, Zheng Shen, Qiong Zhou, Chao-Chun Zou

**Affiliations:** 1 The Children’s Hospital of Zhejiang University School of Medicine, No. 3333 Binsheng Road, Hangzhou, 310051 China; 2 Hangzhou children’s Hospital, No. 195 Wenhui Road, Hangzhou, 310000 China

**Keywords:** Prader–Willi syndrome, Growth hormone, Body composition, Length, Height, Carbohydrate metabolism, Spinal deformity

## Abstract

**Background:**

Prader–Willi syndrome (PWS) is a rare and multisystemic genetic disorder that is characterized by severe hypotonia, hyperphagia, short stature, and global developmental delay. Although early recombinant human growth hormone (rhGH) treatment has been proven to rescue some symptoms and bring additional benefits to PWS patients, studies in patients under 2 years old are scarce. Thus, this study aims to investigate the effectiveness and safety of rhGH treatment for young children.

**Methods:**

A total of 96 genetically confirmed Chinese PWS infants or toddlers (47 males) followed between 2013 and 2022 were retrospectively analyzed. Sixty-five infants (early treatment group) started rhGH treatment during their first year, and 31 toddlers (later treatment group) started at the age of 1–2 years. Auxological parameters, carbohydrate metabolism parameters, thyroid function, liver function, insulin-like growth factor-1 (IGF-1), and radiographs were acquired before the initiation of the treatment and every 3–6 months thereafter. Height/length, weight, and weight for height were expressed as standard deviation scores (SDSs) according to WHO child growth standards.

**Results:**

The mean SDS of length/height in the early treatment group was significantly higher than that in the later treatment group throughout the observation period (all *P* < 0.001). The change in the length SDS between the two groups at 1 year old and 4 years old was 1.50 (95% CI, 0.88–2.13) and 0.63 (95% CI, 0.16–1.10), respectively. Compared to the later treatment group, the weight SDS in the early treatment group increased by 0.94 (95% CI, 0.37–1.52) at 1 year old and 0.84 (95% CI, 0.28–1.39) at 2 years old. No statistical significance was found after 2.5 years of age. No significant differences were observed in IGF-1, incidence of liver dysfunction, hypothyroidism or spinal deformity between the two groups.

**Conclusions:**

rhGH treatment improved growth and body composition in infants and toddlers. Furthermore, an early start of rhGH treatment is expected to have more efficacy than the later treatment group without an increase in adverse effects.

## Introduction

Prader–Willi syndrome (PWS) is a rare genetic disorder resulting from the underexpression of imprinted genes within the chromosome 15q11-q13 region [1, 2]. The prevalence of PWS is approximately 1 in 10,000–30,000 newborns [3, 4]. PWS has three main genetic types, including deletion type with deletion of paternal copy of key region that accounts for 65%-70%, maternal uniparental disomy (mUPD) for chromosome 15 that accounts for 20%-30%, and the imprinting center defect (ID) that includes the imprinting center deletion and epimutation, accounting for less than 5%. Translocation in the region of chromosome 15q11-q13 or small deletions in the key gene of PWS were also reported in very rare cases [5–11]. The clinical presentations vary with age, impacting multiple systems, with the endocrine system being the most affected in PWS [6]. Growth hormone (GH) deficiency (GHD) is present in most cases and may be associated with hypotonia after birth, short stature, increased fat mass, and decreased movement and energy expenditure [12, 13].

Recombinant human GH (rhGH) treatment has been suggested for adults and children with PWS. GH treatment will improve growth body composition, muscle strength, respiratory function, and even psychomotor development. Early rhGH treatment has been found to improve feeding difficulty of infants and cognition of young children with PWS [14–20] which suggests it is better to start treatment before 2 years of age. The timing of starting GH treatment is still controversial (e.g., less than 12 months or 24 months) [21]. Several authors recommended that starting rhGH therapy as early as possible (approximately 3–6 months of age) resulted in additional benefits [22–24]. However, large-sample studies in patients treated at an early age and comparisons of the efficacy of rhGH treatment at different ages are still lacking. Therefore, this study aimed to investigate the effect and safety in Chinese PWS patients aged less than 2 years, especially starting from infancy.

## Material and methods

### Subjects and grouping

This study is part of a project started by the PWS Research Group from the Children’s Hospital of Zhejiang University School of Medicine. In the PWS Research Group and PWS Care & Support Center (an association of PWS patients and parents) register system, 1491 Chinese patients with genetically confirmed PWS were registered until Dec. 2021. A total of 84 subjects were excluded due to a lack of detailed data. Among 1407 PWS patients, 688 (48.90%) had accepted rhGH treatment.

Only 170 patients had correct initiation time of rhGH treatment. Fifteen patients had interrupted rhGH treatment. Among the 170 patients, 86 (50.59%) PWS infants accepted rhGH therapy early in infancy. A total of 65 patients were followed up to 4 years old as the early treatment group, while 21 patients dropped out for unknown reasons. Another 36 patients (approximately 21.17%) started rhGH treatment between 1 and 2 years as the later treatment group. Among them, 31 were followed up to 4 years old. Approximately 19.41% of subjects (33/170) initiated rhGH therapy after 2 years of age.

**Fig. 1:**
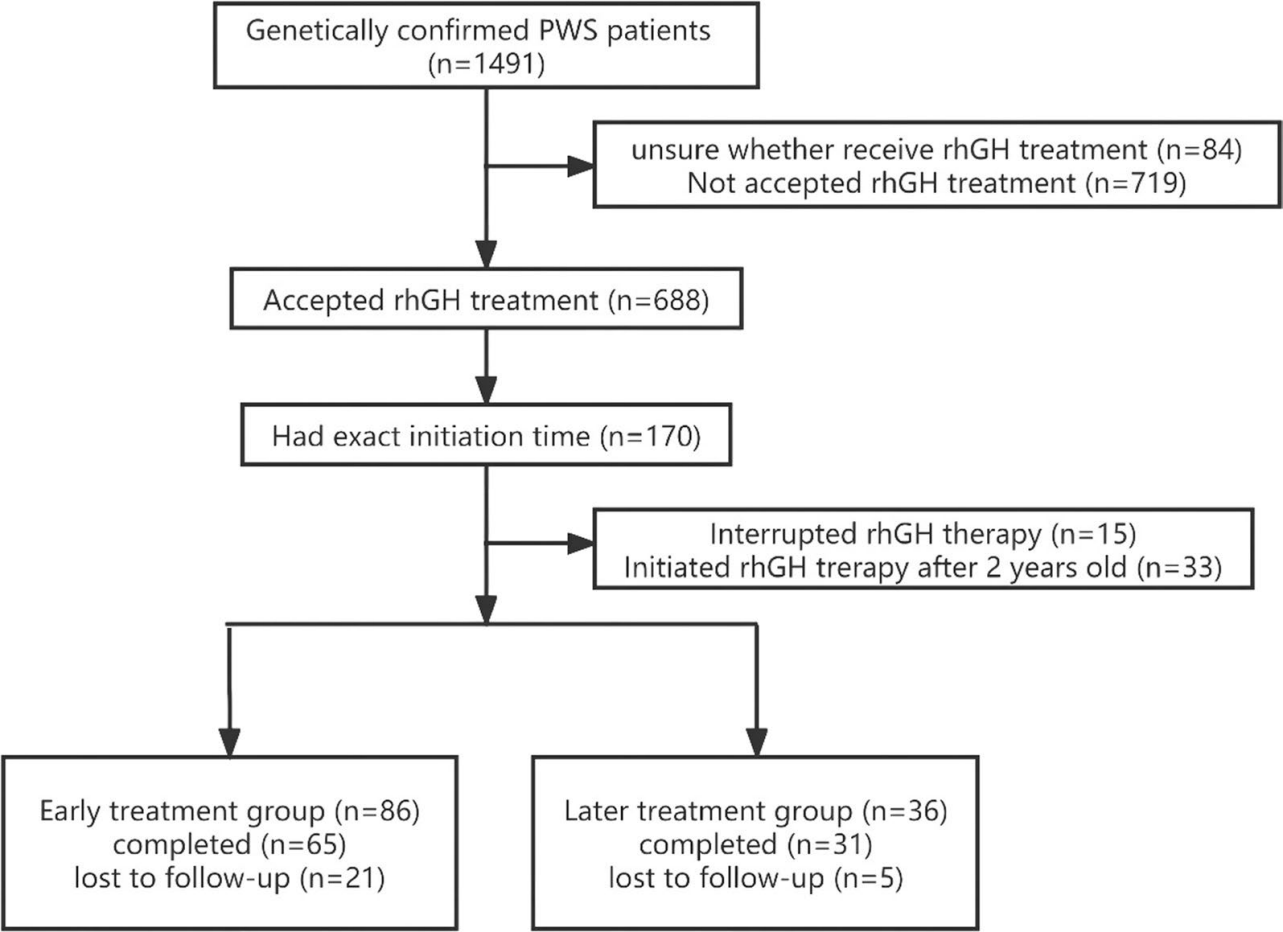
Flow chart of the study population

Finally, 96 PWS pediatric patients were included in this study (Fig. 1), including 65 patients in the early treatment group and 31 patients in the later treatment group. All patients were genetically confirmed to have PWS, including 63 (65.62%) deletion types, 22 (22.92%) nondeletion types, and 11 (11.46%) unknown diagnosed with methylation-sensitive polymerase chain reaction (MS-PCR). rhGH treatment in these patients started at the age of 2 months to 2 years and was not interrupted to 4 years old. The differences in auxological parameters, carbohydrate metabolism, and safety between these two groups of PWS pediatric patients were compared.

The study protocol was approved by the Ethics Committee of the Children's Hospital of Zhejiang University School of Medicine. Informed consent was obtained from all parents or the patients registered in the PWS Registry.

### Follow-up

The dosage of rhGH administered to PWS pediatric patients ranged from 0.46–1.04 mg/m^2^/d with a median of 0.70 (0.20) mg/m^2^/d and was slightly adjusted by professional pediatric endocrinologists according to height, weight and insulin-like growth factor 1 (IGF-1), which kept IGF-1 levels within a 2 standard deviation score (SDS) [21, 25, 26]. No significant difference in rhGH dose was noted between these two groups (P > 0.05). Physical examination, fasting blood samples, standing spinal coronal and radiographs were collected before rhGH therapy and at the ages of 1, 1.5, 2, 2.5, 3, 3.5, and 4 years. Only data within 2 months before and after these time points were considered valid data. The follow-up rates of body composition at 1, 1.5, 2, 2.5, 3, 3.5, and 4 years old were 87.69%, 87.69%, 84.62%, 80.00%, 72.31%, 58.46%, and 47.69% for the early treatment group and 74.19%, 83.87%, 96.77%, 90.32%, 83.87%, 70.97%, and 64.52% for the later treatment group, respectively, without a significant difference.

### Anthropometry

Height/length was measured in a standing/supine position to the nearest 1 mm. Weight was assessed under fasting conditions to the nearest 0.1 kg. SDS for height/length, weight, and weight for height were adjusted for sex and age according to WHO child growth standards (www.who.int).

### Laboratory assessments

Fasting blood samples were collected approximately every 3–6 months for the measurement of fasting glucose (FG) and fasting insulin (FI), IGF-1, thyroid function including thyroid stimulating hormone (TSH), thyroxine (T4), free thyroxine (fT4), triiodothyronine (T3), free triiodothyronine (fT3), and liver function including aspartate transaminase (AST), alanine aminotransferase (ALT). Fasting blood samples were collected at least 3 h after feeding in infancy and overnight after 1 year of age. Insulin resistance (IR) was assessed by the homeostasis model assessment of IR (HOMA-IR), which was defined as [FI (μIU/mL) × FG (mg/dL)]/405 [27]. Due to different kinds of assays in measuring IGF-1, the IGF-1 level was evaluated by age-matched reference values supplied by corresponding manufacturers. The diagnosis of hypothyroidism was made with decreased fT4 (fT4 < 9.01 pmol/L) or increased TSH (> 4.94 mIU/L) 2 times. Liver dysfunction was diagnosed as ALT > 50 U/L and/or AST > 60 U/L without hemolysis in blood samples.

### Spinal radiographs

Standing anteroposterior and later spinal radiographs were evaluated by professional pediatric orthopedic surgeons during the observation period. Spinal deformities, including scoliosis, kyphosis, and kyphoscoliosis, were diagnosed by professional pediatric orthopedic surgeons. The Cobb angle was the curve between the most tilted cephalad and the inferior end plate of the most caudad vertebra. Scoliosis was defined as Cobb angle > 10 degrees.

### Statistical analysis

Statistical analysis was performed by using the Statistical Package for Social Sciences (SPSS Version 21.0, Chicago, IL). Normality was tested by the Shapiro–Wilk test. Normally distributed measurement data are presented as the mean ± SD, while nonnormally distributed measurement data are presented as the median (interquartile range, IQR) depending on the distribution of the data. The homogeneity of the baseline data was assessed by an independent sample t test. Nonparametric tests were performed if needed. Considering multiple testing, the generalized estimated equation (GEE) model followed by Sidak’s post hoc test was performed to evaluate differences in growth and IGF-1 between the early and later treatment groups. According to the QIC value, the auto regression structure was used in this study. In the GEE model, auxological and metabolism parameters were presented as the outcome, age and group were considered the main effect, and the interaction effect of the group by age was also assessed. A significant interaction effect of the group by age suggests differential rates of change in the parameters between the two groups. In addition, the differences in sex and gene subtype were evaluated. The chi-square test was employed to analyze the enumeration data (e.g., sex, genotype, incidence of spinal deformity, and hypothyroidism) between the two groups. A *P* value less than 0.05 was considered statistically significant.

## Results

### Sample description and baseline characteristics

The early treatment group included 29 males and 36 females. Their age of rhGH treatment ranged from 2 to 11 months, with a median age of 6 months. The later treatment group consisted of 18 males and 13 females. Their age of rhGH treatment ranged from 12 to 24 months, with a median of 17 months. The difference in sex was not significant between the two groups (χ^2^ = 1.52, *P* = 0.22).

There were 42 patients (64.62%) with the deletion type, 15 (23.08%) with the nondeletion type, and 8 (12.31%) with undefined types in the early treatment group. There were 21 patients (67.74%) with the deletion type, 7 (22.58%) with the nondeletion type, and 3 (9.68%) with undefined types in the later treatment group. There was no significant difference in genotypes between these two groups (χ^2^ = 0.16, *P* = 0.92).

The mean baseline height/length SDS in the early and later treatment groups were −1.10 ± 1.36 and −1.42 ± 1.54, respectively, without statistical significance (t = 1.04, *P* = 0.30). The baseline weight SDS in the early treatment group was less than that in the later treatment group (−1.81 ± 1.02 vs. −1.24 ± 1.12, t = 2.46, *P* = 0.02). The baseline weight-for-length SDS was -1.58 (2.26) in the early treatment group and −0.94 (1.36) in the later treatment group, without statistical significance (Z = 1.02, *P* = 0.31).

FG and FI were obtained from 32 patients (15 males, 17 females), including 19 in the early treatment group and 13 in the later treatment group. Sex (*P* = 0.28) and genotype (χ^2^ = 0.96, *P* = 0.81) were balanced between these two groups. At baseline, no differences in FG (Z = 0.39, *P* = 0.71), FI (Z = 0.82, *P* = 0.43), or HOMA-IR (Z = 0.68, *P* = 0.52) were found between these two groups, as shown in Table 1.

**Table 1:** Subject demographics and baseline characteristics

	Early treatment group (n = 65)	Later treatment group (n = 31)	t/Z/χ^2^	*P* value
*Subject demographics (n = 96)*				
Gender (male/female)	65 (29/36)	31 (18/13)	1.52	0.218
Age at start treatment (years)	0.50 (0.42)	1.42 (0.67)	7.91	< 0.001
Genotype (deletion/nondeletion/unknown)	65 (42/15/8)	31 (21/7/3)	0.16	0.923
*Auxological parameters (n = 96)*				
Height/length SDS	−1.10 ± 1.36	−1.42 ± 1.54	1.04	0.300
Weight SDS	−1.81 ± 1.02	−1.24 ± 1.12	2.46	0.016
Weight for height SDS	−1.58 (2.26)	−0.94 (1.36)	1.02	0.306
*Carbohydrate metabolism (n = 32)*				
Gender (male/female)	19 (7/12)	13 (8/5)		0.280*
Genotype (deletion/nondeletion/unknown)	19 (15/3/1)	13 (10/3/0)	0.96	0.808
Fasting insulin (μIU/mL)	3.50 (2.38)	3.50 (3.29)	0.82	0.426
Fasting glucose (mmol/L)	4.57 (0.19)	4.57 (0.95)	0.39	0.705
HOMA-IR	0.71 (0.53)	0.71 (0.83)	0.68	0.520

The results are given as the mean ± SD or median (IQR)

*Fisher's precision probability test

### Height/length SDS

**Fig. 2:**
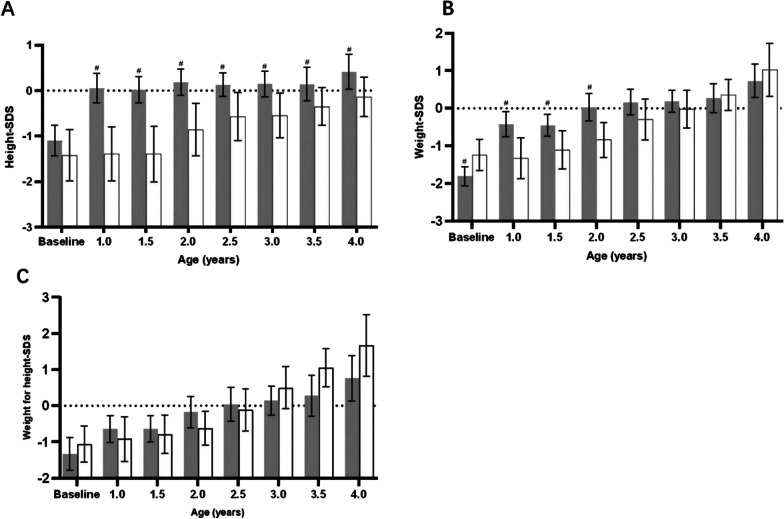
Changes in height SDS (**A**), weight SDS (**B**), and weight for height SDS (**C**) between the early treatment group (shaded) and later treatment group (blank) from baseline to 4 years old. Height-SDS, weight-SDS, and weight for height-SDS were adjusted for sex and age according to WHO child growth standards. Error bars represent means with 95% CI. #, *P* < 0.05, compared with the later treatment group

The generalized estimated equation showed a significant increase in the height/length SDS in both groups over time, as shown in Fig. 2A (time effect χ^2^ = 67.78, *P* < 0.001). The early treatment group was significantly higher than the later treatment group throughout the whole observation period (group effect χ^2^ = 15.54, *P* < 0.001). The height/length SDS at the age of 1 year was significantly higher than that at baseline in the early treatment group (t = 5.38, *P* < 0.001). Nevertheless, in the later treatment group, the height/length SDS was similar to the baseline at 1 year old (t = 1.23, *P* = 0.23). The difference in height/length SDS between the two groups gradually decreased with the development of time. The difference in height/length SDS between the two groups at 1 year old was 1.50 (95% CI, 0.88–2.13), while it was 0.63 (95% CI, 0.16–1.10) and 4 years old (Table 2).

**Table 2:** Height/length, weight, and weight for height between the two groups on the GEE models

	B	95%Cl	*P* value
*Height/length SDS*			
Later treatment group	−1.50	(−2.13, −0.88)	< 0.001
2 years old (early treatment group)	0.17	(−0.07, 0.41)	0.165
3 years old (early treatment group)	0.30	(0.02, 0.58)	0.037
4 years old (early treatment group)	0.49	(0.17, 0.82)	0.003
Later treatment group × 2 years old	0.52	(0.02, 1.01)	0.042
Later treatment group × 3 years old	0.76	(0.27, 1.26)	0.003
Later treatment group × 4 years old	0.88	(0.30, 1.45)	0.003
*Weight SDS*			
Later treatment group	−0.94	(−1.52, −0.37)	0.001
2 years old (early treatment group)	0.48	(0.23, 0.73)	< 0.001
3 years old (early treatment group)	0.88	(0.60, 1.16)	< 0.001
4 years old (early treatment group)	1.28	(0.92, 1.64)	< 0.001
Later treatment group × 2 years old	0.11	(−0.36, 0.57)	0.648
Later treatment group × 3 years old	0.66	(−0.01, 1.33)	0.052
Later treatment group × 4 years old	1.18	(0.37, 1.98)	0.004
*Weight for height SDS*			
Later treatment group	−0.33	(−0.97, 0.32)	0.324
2 years old (early treatment group)	0.50	(0.14, 0.86)	0.006
3 years old (early treatment group)	1.02	(0.63, 1.40)	< 0.001
4 years old (early treatment group)	1.47	(1.00, 1.95)	< 0.001
Later treatment group × 2 years old	−0.15	(−0.77, 0.48)	0.652
Later treatment group × 3 years old	0.47	(−0.38, 1.32)	0.281
Later treatment group × 4 years old	1.16	(0.14, 2.19)	0.026

The reference group for each variable is early treatment group, 1 year old (early treatment group), early treatment group × age

### Weight and weight for height/length SDS

The GEE model showed a significant time effect in weight SDS (χ^2^ = 94.64, *P* < 0.001). However, there were no significant main effects of group (χ^2^ = 3.16, *P* = 0.08) or group by age interaction (χ^2^ = 11.50, *P* = 0.07). The difference in weight SDS between the two groups at 1 year old was 0.94 (95% CI, 0.37–1.52) and 0.84 (95% CI, 0.28–1.39) at 2 years old. However, at the age of 2.5 years old, the difference in weight SDS was 0.46 (95% CI, −0.14 to 1.06), without statistical significance. From 2.5 years old, the weight SDS did not show significant differences between the early and later treatment groups (*P* = 0.14), as shown in Fig. 2B.

No significant differences in group effect (χ^2^ = 0.09, *P* = 0.77) or group by age interaction effect of weight and height SDS (χ^2^ = 10.23, *P* = 0.12) were observed throughout the whole observation. Only at the age of 4 years old was the weight for height slightly increased by 0.83 (95% CI, −0.01 to 1.68, *P* = 0.052) in the later treatment group compared to the early treatment group (Fig. 2C and Table 2).

### Serum IGF-1

Serum IGF-1 levels were obtained from 37 children, including 25 in the early treatment group and 12 in the later treatment group. The kinds of assays that measure serum IGF-1 were different in different laboratories in this study. Thus, compared with age-matched reference intervals supplied by corresponding manufacturers, the rank of IGF-1 level was divided into high (> mean + 1SD), normal (mean ± 1SD), and low (< mean-1SD). No significant effects of group (χ^2^ = 0.47, *P* = 0.49), gender (χ^2^ = 0.44, *P* = 0.51), or genotype (χ^2^ = 0.96, *P* = 0.62) were observed by the GEE model. The time effect was strong (χ^2^ = 22.66, *P* < 0.001). At 1 year old, 9 individuals had lower levels of IGF-1 than the normal range, while only one was 4 years old, with a significant difference (*P* < 0.001).

### Carbohydrate metabolism

**Fig. 3:**
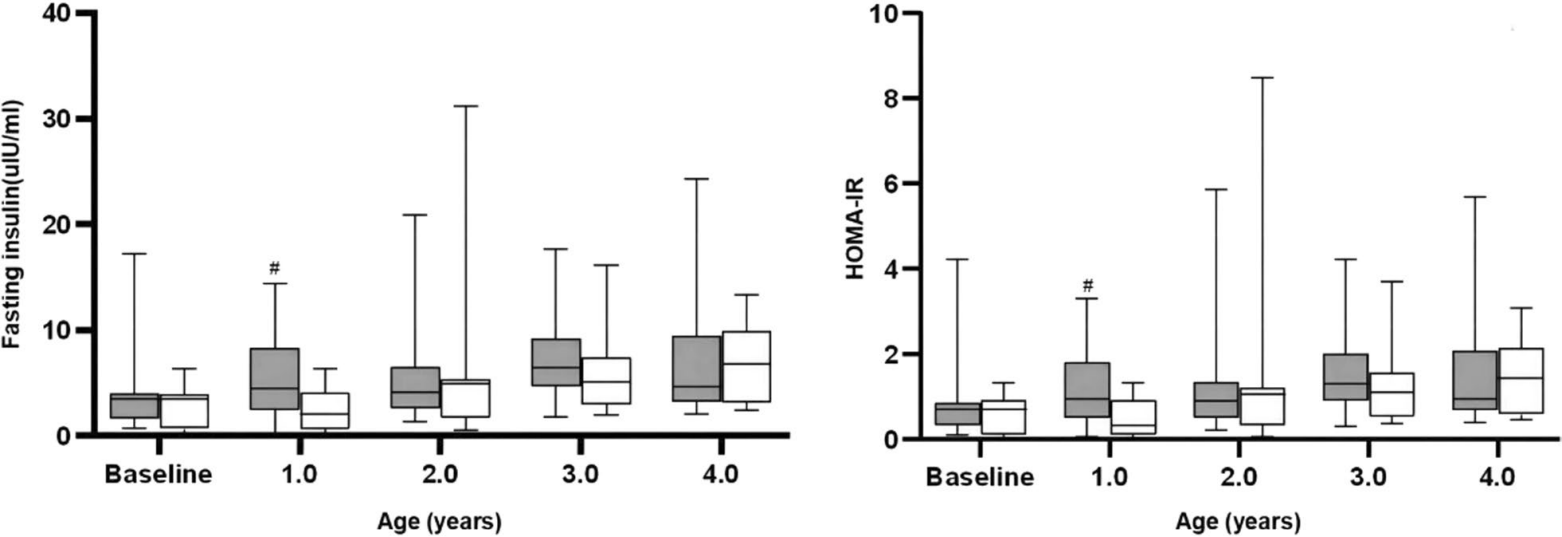
Changes in fasting insulin and HOMA-IR between the early treatment group (shaded) and later treatment group (blank) from baseline to 4 years old. The lower and upper bounds are the 25th percentile and the 75th percentile, respectively. The horizontal line in the box shows the median. #, *P* < 0.05, compared with the later treatment group

Fasting blood samples were obtained from 32 patients (15 males, 17 females), including 19 in the early treatment group and 13 in the later treatment group. At the age of 1 year, the FIs in the early treatment group and later treatment group were 4.47 (5.90) and 2.00 (3.50), respectively, with a significant difference (Z = 2.37, *P* = 0.016). Moreover, HOMA-IR in the early treatment group was significantly higher than that in the later treatment group at 1 year old (Z = 2.33, *P* = 0.018). However, the effect was reserved when the later treatment group started rhGH treatment, with no statistical significance during the course of the study. No significant difference in FG was observed between the two groups throughout the observation period (Fig. 3). In the first or second year of treatment, 3 patients (15.79%) had FG > 5.6 mmol/L in the early treatment group, while 2 patients (15.38%) had FG > 5.6 mmol/L in the later treatment group. The highest FG in our study was 6.32 mmol/L. However, their FG eventually returned to the normal range in the following time. No cases of diabetes were observed during the whole observation period.

### Thyroid and liver function

Hypothyroidism was reported in 13 patients (13.54%), and 3 patients were diagnosed before rhGH treatment. Among the 10 patients diagnosed with hypothyroidism during rhGH treatment, 9 (13.85%) were in the early treatment group, while 1 (3.23%) was found in the later treatment group. No significant difference (χ^2^ = 1.53, *P* = 0.22) was found, as shown in Table 3. Of them, 9 patients had accepted thyroxine treatment during the observation period. Hyperthyroidism was not noted throughout the observation period.

**Table 3:** Adverse events of the patients between groups

	Early treatment group	Later treatment group	χ^2^	*P* value
Hypothyroidism	9 (13.85%)	1 (3.23%)	1.53	0.217
Liver dysfunction	2 (3.08%)	0 (0.00%)		1.000*
Spinal deformities	15 (23.08%)	6 (19.35%)	0.17	0.680

*Fisher's precision probability test

Liver dysfunction was noted in seven patients, and ALT ranged from 54 U/L to 190 U/L. Of them, 5 patients were found before accepting rhGH treatment. During rhGH treatment, 2 patients (3.08%) were diagnosed in the early treatment group, and no patient was found in the later treatment group, without a significant difference (*P* = 1.00), as shown in Table 3. Three patients had accepted liver-protected drugs, and ALT in all patients returned to the normal range in the following time.

### Spinal deformity and others

In this series, spinal deformities were noted in 21 (21.88%) patients by spine radiography. Of them, 15 (23.08%) patients were in the early treatment group, and 6 (19.35%) were in the later treatment group without statistical significance (χ^2^ = 0.17, *P* = 0.68), as shown in Table 3. Approximately 27.66% (13/47) of patients with spinal deformities were male, and 16.33% (8/49) were female, without a significant sex difference (χ^2^ = 1.80, *P* = 0.18). No allergic reaction or injection site infection was reported during the rhGH treatment.

## Discussion

Among the registered PWS patients in China, 48.9% (688/1407) accepted rhGH treatment, which was comparable to the Italian National survey for PWS patients from 1986 to 2006 [28]. The relatively “higher” ratio of rhGH treatment may be associated with the fact that over 70% registered PWS younger than 5 years and diagnosed in the past 5 years (unpublished data). In fact, the ratio of rhGH treatment may be lower than 48.9%, as rhGH treatment may be lower in unregistered PWS patients. In Hong Kong, only 1.79% of PWS patients (1/56) had accepted rhGH treatment in the Chinese PWS cohort from 1995 to 2010 [29]. A considerable cost and incurability might result in a low ratio of accepting rhGH therapy [30].

Moreover, the median age at rhGH treatment start in our project ranged from 1 to 201 months with a median of 10.5 months, which was significantly younger than that in the United States and Europe (approximately 4 to 5 years) [31]. This may also be associated with the fact that most patients in our cohort were less than 10 years old. These results implied significant improvements in the diagnosis and management of PWS in recent years. However, misdiagnosis, misdiagnosis of PWS, and less rhGH treatment might have been common in China 10 years ago.

To our knowledge, this is the first large sample study of rhGH treatment in younger children in China. In our longitudinal follow-up, rhGH treatment significantly improved the height/length SDS from baseline in both early (from −1.10 ± 1.36 SDS at baseline to 0.42 ± 1.10 SDS at 4 years old) and later treatment groups (from −1.42 ± 1.54 SDS at baseline to −0.14 ± 0.85 SDS at 4 years old), which was consistent with previous reports [32, 33]. At the age of 1 year, the height/length SDS in the later treatment group was similar to the baseline height/length SDS, which was comparable with an untreated Chinese PWS population[34]. Notably, the height/length in the early treatment group was normalized to an SDS of 0.06 ± 1.53 at 1 year old, which was significantly greater than that in the untreated later treatment group. Although the height velocity increased dramatically in the first rhGH treatment year in both the early and later treatment groups, the height/length SDSs in the early treatment group were all different from those in the later treatment group at various age points. At 4 years of age, the mean height (0.42 ± 1.10 SDS) in the early treatment group was slightly higher than normal but shorter in the later treatment group (-0.14 ± 0.85 SDS). Several studies reported that initiation of rhGH treatment in prepuberty could completely normalize stature after long-term therapy[35–37]. Our results showed that early rhGH treatment may reach the normalization height earlier than later treatment. Whether early rhGH treatment significantly improved the final height required further longer follow-up, as this cohort was only followed up to 4 years old.

It was notable that the later treatment group was heavier than the early treatment group at baseline, which may be associated with parents being more willing to use rhGH early in patients with poorer suck and feeding difficulty in infancy. The weight for height in the early treatment group was slightly lower than that in the later treatment group at 4 years old, which suggested that rhGH treatment could increase lean muscle mass and decrease body fat in PWS patients [37, 38]. However, it is worth noting that weight in the early treatment group was higher than that in the later treatment group before the age of 2.5 years, especially the weight almost reaching the normal range in the early treatment at the age of 1 year. These results suggested that rhGH treatment had a “two-way regulation” function for body weight. It increases muscle mass, improves feeding difficulties, and then increases energy intake [39]. Both muscle mass and energy intake increase may improve the weight and weight for height in infancy. Conversely, rhGH may reduce fat mass by increasing the metabolic rate to reduce obesity after 2–2.5 years of age. Although the differences in weight and weight for height were not significant, we noted that the weight and weight for height were closer to the normal range in the early treatment group. In addition, it may also be associated with the fact that parents in the early treatment group have better compliance and stronger aspiration to control their children’s body weight. Weight intervention programs, including diet control and physical activity, may cause body composition improvement [40]. Thus, early diagnosis and treatment, as well as education for parents, are important for PWS management.

During the follow-up, allergic reactions and injection site infections were not reported. It was notable that FI in the early treatment group was higher than that in the later treatment group during the first treatment year. This implied that IR may be present during rhGH treatment. Fortunately, FI and HOMA-IR were stable, and no differences were observed between the two groups in the following years. Moreover, FG was not different between the two groups, and no cases of diabetes were observed, although transient hyperglycemia was noted in 5 patients. This was similar to most previous studies about adult or older children [41–43]. However, different from those studies in which rhGH treatment had no effects on glucose homeostasis in PWS patients [37, 44–46]. Thus, although early rhGH treatment did not increase the IR, glucose homeostasis should be evaluated regularly for PWS patients treated with rhGH.

Although no hyperthyroidism was found, hypothyroidism was reported in 13 patients (13.5%) during rhGH treatment, which was similar to the prevalence in the Italian multicenter study [47]. Moreover, liver dysfunction was found only in 2 patients (3.08%), and ALT returned to the normal range in the following time. No significant differences in the prevalence of hypothyroidism or liver dysfunction were observed between the two groups. Oto et al. also reported that thyroid function did not significantly change after 2 years of rhGH treatment [48]. However, the prevalence was statistically higher in the early treatment group. Therefore, thyroid and liver function should be routinely monitored during rhGH treatment, especially in infancy.

The incidence of spinal deformities was as high as 21.88%, which was similar to previous studies [49, 50]. Whether it is associated with rhGH treatment is unknown, as nonrhGH treatment data were not included in this study. However, there were no significant differences in the incidence of spinal deformities between the two groups, which was similar to a previous study of older children [51, 52]. It was reported that older and higher BMI PWS patients presented a higher incidence of scoliosis [53]. Hence, scoliosis was still periodically monitored during rhGH treatment [21]. Nevertheless, scoliosis should not be considered a contraindication of rhGH therapy in PWS patients.

There were some limitations in our study. First, some follow-up data were lacking at some time points and were lost to follow-up, as most patients are from all over the country and have relatively poor compliance in China. Second, as most untreated PWS patients in the PWS Research Group missed follow-up, we could not compare auxological parameters with those of untreated patients. Third, rhGH treatment was also reported to improve psychomotor development in some literature, which was not documented in our study. Fourth, lean body mass and percent body fat were not measured.

In summary, this longitudinal study showed that younger children with PWS may benefit greatly from rhGH treatment and that early rhGH treatment had a more favorable outcome in height/length and body composition. Thyroid dysfunction, liver dysfunction, and scoliosis should be monitored during rhGH treatment, and the risk of adverse effects will not increase for PWS patients starting rhGH therapy in infancy.

## Data Availability

Unpublished data in this manuscript will be shared by request from any qualified investigator.

## Abbreviations

PWS: Prader–Willi syndrome
rhGH: Recombinant human growth hormone
IGF-1: Insulin-like growth factor-1
SDS: Standard deviation score
FG: Fasting glucose
FI: Fasting insulin
TSH: Thyroid stimulating hormone
T4: Thyroxine
fT4: Free thyroxine
T3: Triiodothyronine
fT3: Free triiodothyronine
AST: Aspartate transaminase
ALT: Alanine aminotransferase
